# Timing-Dependent Protection of Swimming Exercise against d-Galactose-Induced Aging-Like Impairments in Spatial Learning/Memory in Rats

**DOI:** 10.3390/brainsci9090236

**Published:** 2019-09-14

**Authors:** Xue Li, Lu Wang, Shuling Zhang, Xiang Hu, Huijun Yang, Lei Xi

**Affiliations:** 1 Department of Human Anatomy, West China School of Preclinical and Forensic Medical Institute, Sichuan University, Chengdu 610041, China; 2 Department of Human Kinesiology, School of Sports Medicine and Health, Chengdu Sport University, Chengdu 610041, China; 3 Department of Morphology Laboratory, Chengdu Medical College, Chengdu 610083, China; 4 Department of Internal Medicine, Virginia Commonwealth University, Richmond, VA 23298-0204, USA; lxi@vcu.edu

**Keywords:** aging, exercise, hippocampus, learning, memory, neurodegeneration, neuroprotection

## Abstract

This study was designed to investigate beneficial effects of swimming exercise training on learning/memory, synaptic plasticity and CREB (cAMP response element binding protein) expression in hippocampus in a rat model of d-galactose-induced aging (DGA). Eighty adult male rats were randomly divided into four groups: Saline Control (group C), DGA (group A), Swimming exercise before DGA (group S1), and Swimming during DGA (group S2). These four groups of animals were further divided into Morris water maze training group (M subgroup) and sedentary control group (N subgroup). Spatial learning/memory was tested using Morris water maze training. The number and density of synaptophysin (Syp) and metabotropic glutamate receptor 1 (mGluR1) in hippocampal dentate gyrus area, CREB mRNA and protein expression and DNA methylation levels were determined respectively with immunohistochemistry, western blot, real-time PCR, and MassArray methylation detection platform. We found that compared with group C, DGA rats showed aging-like poor health and weight loss as well as hippocampal neurodegenerative characteristics. Exercise training led to a time-dependent decrease in average escape latency and improved spatial memory. Exercise training group (S2M) had significantly increased swim distance as compared with controls. These functional improvements in S2M group were associated with higher Syp and mGluR1 values in hippocampus (*p* < 0.01) as well as higher levels of hippocampal CREB protein/mRNA expression and gene methylation. In conclusion, swimming exercise training selectively during drug-induced aging process protected hippocampal neurons against DGA-elicited degenerative changes and in turn maintained neuronal synaptic plasticity and learning/memory function, possibly through upregulation of hippocampal CREB protein/mRNA and reduction of DGA-induced methylation of CREB.

## 1. Introduction

The signaling mechanisms related to learning/memory function have been a focal topic in the field of neuroscience [1,2]. The role of hippocampus in the process of memory formation has been increasingly realized. The synapses in hippocampus can enhance conduction in high frequency stimulation and play an important role in memory. It has been suggested that aging impairs cerebrovascular plasticity and subsequently leads to cerebral hypoperfusion, which synergistically accelerates aging-associated cognitive dysfunction, impaired neuronal plasticity, and loss of learning ability and memory [3]. In the past decades, physical activity has been well recognized as a factor to improve brain memory function. It was demonstrated that long-term mild exercise training enhances hippocampus-dependent memory in adult rats [4]. These authors showed that six weeks of continuous mild exercise training enhanced memory assessed with a Morris water maze task, whereas no such an improvement was found in the rats under intensive exercise training, which led to general adaptive syndrome (including adrenal hypertrophy, thymic atrophy and hypercorticosteronemia) [4]. Most recently, the same research group also showed that in young human subjects a single 10-min bout of light-intensity exercise resulted in rapid enhancement in pattern separation and increased functional connectivity between hippocampal DG/CA3 (dentate gyrus/Cornu Ammonis 3 subregion) and cortical regions (i.e., parahippocampal, angular, and fusiform gyri) [5]. In addition to improvements in neuronal plasticity and neurogenesis, exercise can also improve cerebrovascular plasticity and cerebral blood flow [6].

A sub-acute rodent model of chemically induced neuronal changes with d-galactose simulating the natural and accelerated senescence has been used as a valid model in aging brain research [7,8]. Interestingly, several previous studies reported that moderate aerobic exercise intervention protected against d-galactose-induced aging (DGA) [9,10,11]. Treadmill exercise reduced cognitive deficits in the DGA rats and suppressed their hippocampal oxidative stress and amyloid production [11]. In addition, forced, moderate-intensity treadmill exercise suppresses apoptosis by increasing nerve growth factor (NGF) levels and activating phosphatidylinositol 3-kinase (PI3K) signaling in the hippocampus of DGA rats [9]. Similar findings were recently obtained in a mouse model of DGA, in which moderate exercise prevented neurodegeneration in DGA mice [10], along with upregulation of neuroprotective proteins such as Bcl-2 and brain-derived neurotrophic factor (BDNF).

cAMP/PKA/CREB signaling pathway is directly involved in formation of learning and memory. CREB (cAMP response element binding protein) especially plays a central role in terminating and intersecting cell signaling in the central nervous system and in turn regulating its target genes that are involved in the regulation of neuronal cell cycle, induction and differentiation of neurons, and modifying synaptic plasticity. In addition, DNA methylation pattern is also affected by brain aging. For example, DNA methylation in the promoter region of BDNF may lead to a decline in learning and cognition. On the other hand, exercise intervention, serving as an exogenous stimulus for improving learning and cognitive ability [10,12,13,14], may also lead to changes in the methylation level of cellular genomes as well as selective modification in gene expression such as CREB. However, how exercise training affects *CREB* gene expression in brain and whether it is correlated with the hippocampal synaptic plasticity changes remain largely uncertain. Similarly, whether exercise could affect histone modification on cerebral *CREB* gene is not clear.

In addition, to our best knowledge, most of the previous aging animal studies on exercise and learning/memory function were conducted using forced treadmill run as the mode of exercise [9,10,11]. It remains unknown if another major modality of physical exercise—swimming would afford similar neuroprotective effects. It has been well recognized in exercising rodents that with a comparable exercise intensity in the same groups of animals, swimming and treadmill running would produce significantly different cardiovascular and hormonal responses [15,16] as well as distinguishable profiles in stress biomarkers [17]. Hence the possibilities of similar or different effects of swimming training on neuronal function in aging rodent require further thorough investigation.

Therefore, to address the abovementioned unsolved issues, the present study was designed to investigate the timing-effects of swimming exercise training on learning/memory, synaptic plasticity and *CREB* gene expression and DNA methylation level in the hippocampus of d-galactose-induced aging-like rats. We attempted to provide novel mechanistic insights on how does swimming exercise training delay or slow-down the d-galactose-induced brain structural changes and functional impairments in learning and memory.

## 2. Materials and Methods

### 2.1. Animals and Rat Model of Drug (d-Galactose)-Induced Aging

**Figure 1:**
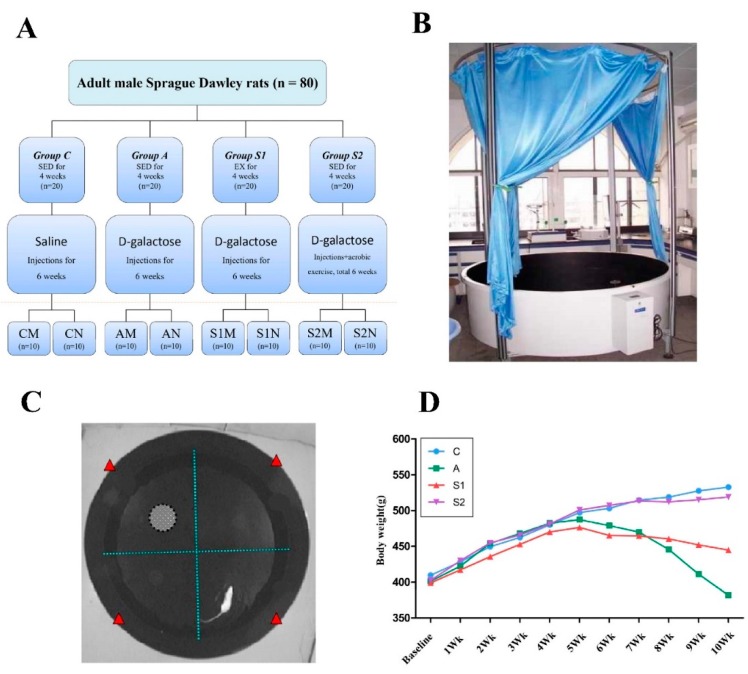
(**A**) provides an illustrative description of the overall experimental group assignment and treatment protocol design. Each treatment group (i.e., group C, A, S1, S2; *n* = 20/group) was randomly divided into two subgroups: one received Morris water maze training (M subgroup, *n* = 10) and the other group had no training (N subgroup, *n* = 10). *Abbreviations*: SED—sedentary; EX—exercise. (**B**) presents a photograph of Morris thermostat water maze system used for the behavioral training and testing experiments. The navigation test was conducted in a platform placed in the fourth quadrant of a round pool (**C**) and the activities of the swimming rats were continuously recorded and timed with a video monitor system. (**D**) shows a 10-week time course of the weekly changes in body weight in the various experimental groups, i.e., group C, A, S1, and S2 (*n* = 20 per group).

A total of 80 male specific pathogen-free Sprague Dawley rats, aged 3 months, and weighing 400 ± 10 g were studied. The rats were purchased from Chengdu Dashuo Biological Technology Co., Ltd. (Chengdu, China). Rats were allowed to adapt to the new environment for one week and then were randomly divided into four groups (*n* = 20 in each group) as follows: Saline control group (Group C), d-galactose injected (DGA) group (Group A), Exercise training before DGA group (Group S1), Exercise training during DGA group (Groups S2). Each group was then randomly divided into two subgroups: one group was given Morris water maze training (M subgroup, *n* = 10 each), while the other group received no training (N subgroup, *n* = 10 each). Experimental protocols for all groups are illustrated in Figure 1A.

The drug-induced sub-acute aging model was established by intraperitoneal injections of d-galactose according to the previously reported methods [8,10,18]. d-Galactose was diluted in saline into a concentration of 5% solution, which was administered intraperitoneally once daily with a volume of 2 mL/kg, achieving the daily dose of 100 mg/kg and continued for six weeks. Group C rats were injected with an equivalent volume of saline only. The animal experiments were carried out in accordance with the U.S. National Research Council Committee for the Update of the Guide for the Care and Use of Laboratory Animals (8th edition, 2011) and the experiment protocol was approved by the Institutional Animal Care and Use Committee of Chengdu Sport University.

### 2.2. Exercise Protocols and Subsequent Tests and Tissue Collections

Swimming was selected as the aerobic exercise mode in a transparent glass jar (160 cm × 60 cm × 110 cm). The water depth was 80 cm, and the temperature was 32 ± 2 °C. All groups, except group C and group A, engaged in a moderate swimming workload before (group S1) or during (group S2) the DGA treatment. As shown in Figure 1A, rats in S1 groups were adapted to swimming for three days prior to beginning the actual experiments, after which consisted of 60 min swimming per day, 6 days a week for 4 weeks. This protocol of swimming exercise under moderate intensity was adopted with modifications from the previous studies in swimming rats, with similar exercise duration and water temperature [16]. Rats in S2 groups performed the same daily swimming training each morning and then received DGA injection in the afternoon daily for 6 weeks. Subsequently, rats in each M subgroup performed the Morris water maze test daily for 7 days, rats in each N subgroup were simply fed for 7 days as the time-matched controls. At the end of the experiments, rats were sacrificed by decapitation using an aseptic sharp scissors, which was recommended as a preferable euthanasia method in considerations of both animal welfare perspective and data quality for obtaining brain tissue samples without unwanted chemical contamination of gaseous or liquid anesthetics [19]. Following craniotomy, brain tissues were quickly removed and the hippocampus was separated. Eight samples from each group were fixed in 4% neutral paraformaldehyde liquid and saved for paraffin embedding. The other samples were stored in liquid nitrogen for molecular biology analysis. Both histology and molecular biology experiments were conducted using the facilities of the Key Laboratory of Transplantation Engineering and Immunology, Sichuan University (Chengdu, China).

### 2.3. Morris Water Maze Behavioral Test

A Morris thermostat water maze system was used for behavioral training and testing in each of the M subgroups. The training and testing included two stages, i.e., “navigation test” and “experimental space exploration”. The navigation test was done on a platform that was placed in the fourth quadrant of a round pool (Figure 1B). The rats were placed into the pool facing any point on the wall. A video system automatically recorded the swimming paths of the rats and counted exactly the time for each rat to find platform. After a rat found the platform, or if a rat failed to find the platform within 2 min (the time limit to consider as latency), the rat was guided to the platform and allowed to rest for 10–20 s, after which the next experiment was performed. Rats underwent training 4 times daily, for six days, and all training sessions were performed in the darkness. Subsequently, the space exploration experiments were done 24 h after the navigation test ended. The platform was removed and the rat was placed in the second quadrant of the pool. The time needed for the rats swam through the area where the platform originally located (i.e., the fourth quadrant) was recorded and the percentage of swimming distance in the fourth quadrant versus the total distance was calculated.

### 2.4. CREB mRNA Expressions in Hippocampus

Total RNA was extracted from appropriate hippocampal tissues using Trizol (Invitrogen, Chengdu, China). Total RNA concentration and purity was determined, and RNA integrity was analyzed using agarose gel electrophoresis. The cDNA was synthesized by reverse-transcription using PrimeScript Reagent Kit (Takara Bio, Inc., Shiga, Japan). The primer sequences of CREB and the reference gene β-actin for real-time PCR amplification were designed and synthesized by TaKaRa Bio Inc. Real-time PCR experiment were performed following the manufacturer’s instructions (Bio-Rad Laboratories, Hercules, CA, USA). After the reactions were completed, the relative gene expressions were calculated using the 2^−ΔΔCT^ method.

### 2.5. Hippocampal CREB Protein Content and β-Actin as Protein Loading Control

Hippocampal tissues were homogenized and placed in ice water for 30 min. The lysates were centrifuged and the supernatant was collected. Protein denaturation was done in a 100 °C water bath for 10 min and protein concentration was determined using a BCA kit (Beyontime Biotechnology, Chengdu, China). The protein samples were separated by SDS-PAGE electrophoresis and transferred to a polyvinylidene difluoride (PVDF) membrane. The PVDF membrane was blocked in 5% BSA at room temperature for 1 h and then incubated with a primary mouse monoclonal antibody for total CREB (1:1000 dilution; ProMab Biotechnologies Inc., Richmond, CA, USA) and kept overnight at 4 °C on a shaker. Next morning the PVDF membrane was washed with TBST buffer and re-incubated with a goat anti-mouse horseradish peroxidase-conjugated secondary antibody (1:2000 dilution; Cell Signaling Technology, Danvers, MA, USA) for 2 h at 37 °C. The membrane was then washed and the protein bands were visualized via enhanced chemiluminescence solution and subsequently exposed to an X-ray film. The optical density of the proteins bands was quantified using a computer image analysis program and the CREB expression level was normalized to β-actin expression (using a mouse monoclonal antibody with 1:1000 dilution; Beijing Golden Bridge Biotechnology Co., Ltd., Beijing, China), which served as the internal control for protein loading.

### 2.6. Epigenetic Markers

The rat *CREB* gene sequences and other related information were obtained from the National Center for Biotechnology Information (NCBI) website. The fragment containing nucleotide 56–470, with concentrated CpG sites, was selected as the target sequence. EpiDesigner software was used to design primers. A Sequenom MassArray methylation detection platform of time of flight mass spectrometry (Liuhe Beijing Genomics Technology Co, Ltd., Beijing, China) was used for detecting the epigenetic markers of *CREB* gene methylation.

### 2.7. Data Analysis

SPSS 19.0 statistical software (IBM, Armonk, NY, USA) was used for statistical analysis. Data were quantitated as Mean ± Standard Deviation (SD) and analyzed by one-way ANOVA followed by Student-Newman-Keuls post-hoc test for multiple comparison between the groups. Statistical significance was set at the level of *p* < 0.05.

## 3. Results

### 3.1. General Health Status of Animals

Group C rats had good mental states, ate normal diets, were agile, had moist, shiny body hair, and healthy bodies. Group A rats suffered from malaise, loss of appetite, low excretion, and drowsiness. Furthermore, they show obvious signs of aging, such as slow movement and hair loss. As a quantitative measure of health status, the weekly changes in body weight was plotted as time course in Figure 1D, which showed that during the 10-week experiment period, group C had a gradual increase in body weight representing a normal growth, whereas group A had a time-dependent decline in body weight starting from the first daily injection of DGA on the 5th week of the protocol and such a weight loss was gradually worsen by the end of 10th week. On the other hand, rats in both S1 and S2 groups maintained their body weight significantly better than group A, whereas S2 group kept their body weight similar to group C, indicating swimming exercise training during DGA treatment essentially blocked DGA induced aging-like body weight loss (Figure 1D). 

### 3.2. Morris Water Maze Navigation Test

**Figure 2:**
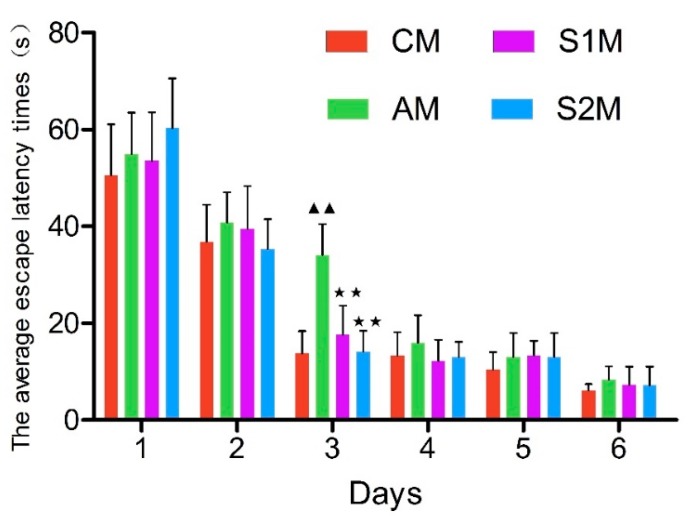
Time-dependent effects of the navigation training on the average escape latency in rats. As the training time increased, the average escape latency gradually decreased in all groups towards a stable spatial memory at Day 6. Data are presented as Mean ± Standard Deviation (SD; *n* = 10/group). The in-graph symbols indicate: ^▲▲^ *p* < 0.01 versus *group CM*; ** *p* < 0.01 versus *group AM*.

The results of the navigation training experiment (Figure 2) showed that the average escape latency of rats in each group gradually decreased following the daily training sessions. The spatial memory of platform location was initially formed on Day 2. A stable spatial memory was formed on Day 3 in Group CM, S1M and S2M, but not Group AM (*p* < 0.01 versus all other groups), indicating the retarded formation of spatial memory in DGA animals. However, this inter-group difference disappeared on Day 4 to Day 6 (Figure 2).

### 3.3. Spatial Exploration

**Figure 3:**
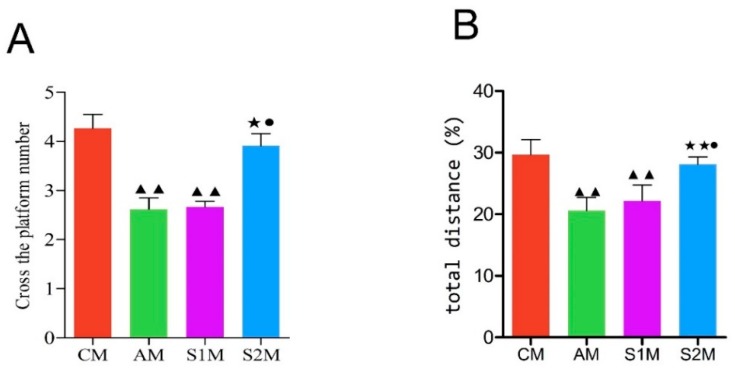
Quantitative assessment of the ability of spatial exploration in rats. (**A**): Time spent to cross the platform in rats of each M subgroup. (**B**): The % ratio of swim distance to the original platform over the total swim distance of the rats in group M. Please note that both the number of times of crossing platform (**A**) and % ratio of swim distance to original platform quadrant versus the total swim distance (**B**) were significantly higher in group CM and S2M, as compared with group AM (*p* < 0.01) or S1M (*p* < 0.05). Data are presented as Mean ± SD (*n* = 10/group). The in-graph symbols indicate: ^▲▲^ *p* < 0.01 versus group CM; * *p* < 0.05 or ** *p* < 0.01 versus group AM; ^●^ *p* < 0.05 versus group S1M.

The number of times of crossing platform and percentage of swim distance to original platform quadrant versus the total distance was highest in CM, followed by group S2M, and significantly higher in CM compared with groups AM, S1M (*p* < 0.01). Group S2M is significantly higher than groups AM, S1M (*p* < 0.05). The percentage of swim distance to original platform quadrant versus the total distance (Figure 3A,B) is significantly higher in group AM (*p* < 0.01) and group S1 (*p* < 0.05).

### 3.4. Immunofluorescence of Synaptophysin (Syp) in Hippocampus

**Figure 4:**
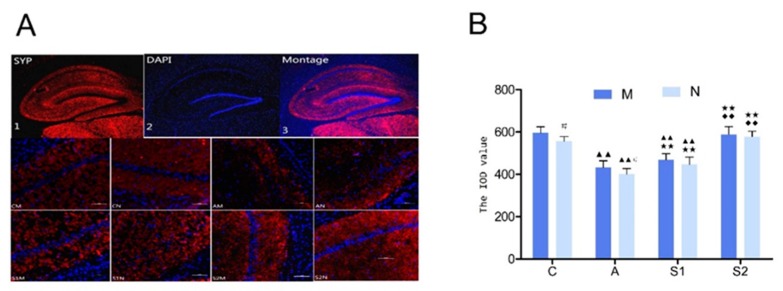
(**A**): Representative images showing the expression levels of synaptophysin (Syp) in the rat hippocampal dentate gyrus (DG) region, which were assessed by immunofluorescent staining and captured by laser confocal scanning microscopy (× 200 magnification; the scale bar indicates a length of 100 µm). The red fluorescence was Syp of Cy3, blue fluorescence was the nuclei of DAPI-labeled hippocampal neurons, and the superimposed image. (**B**): Changes of integral optical density (IOD) levels of Syp in the hippocampus DG region, which were calculated by image processing analysis. Data are presented as Mean ± SD (*n* = 10/group). The in-graph symbols indicate: ^▲▲^ *p* < 0.01 versus *Group C*; ** *p* < 0.01 versus *Group A*; ^◆◆^ *p* < 0.01 versus *Group S1*; ^#^ *p* < 0.05 versus *Group M*.

The integral optical density (IOD) values of Syp in the hippocampus DG region were calculated by image processing analysis using Image pro-plus software (Figure 4B). Compared with group C, the levels of Syp significantly decreased in group A and S1 (*p* < 0.01). To the contrary, compared with group A and S1, the Syp levels significantly increased in group S2 (*p* < 0.01).

### 3.5. Immunofluorescence of mGluR1 in Hippocampus

**Figure 5:**
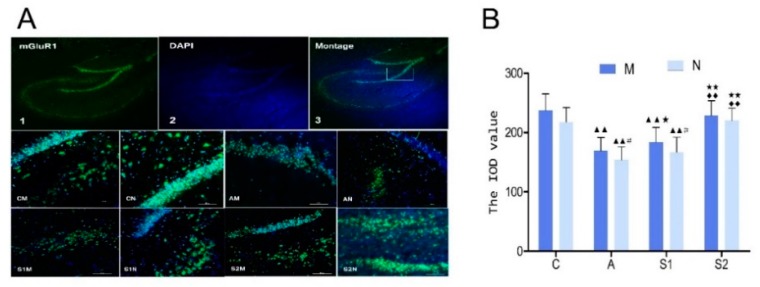
(**A**): Representative images of metabotropic glutamate receptor 1 (mGluR1) in the rat hippocampal dentate gyrus (DG) region. The expression levels of mGluR1 were assessed by immunofluorescent staining and the integral optical density (IOD) values were quantified in the images captured by laser confocal scanning microscopy (× 200 magnification; the length scale bar indicates 100 µm). The red fluorescence was mGluR1 of FITC, blue fluorescence was the nuclei of DAPI-labeled hippocampal neurons, along with the superimposed image. (**B**): Changes of mGluR1 IOD value in rat hippocampus DG region, which were quantified with image vector analysis. Data are presented as Mean ± SD (*n* = 10/group). The in-graph symbols indicate: ^▲▲^ *p* < 0.01 versus *Group C*; * *p* < 0.05 or ** *p* < 0.01 versus *Group A*; ^◆◆^ *p* < 0.01 versus *Group S1*; ^#^ *p* < 0.05 versus *Group M*.

The IOD value of mGluR1 in rat hippocampus DG region (Figure 5B). Compared with group C, group A and group S1 significantly decreased (*p* < 0.01). Group S1 is significantly higher than group A (*p* < 0.05), and group S2 significantly increased (*p* < 0.01). Group S2 significantly increased compared with group S1 (*p* < 0.01). 

### 3.6. Hippocampal CREB Protein and mRNA Expression

**Figure 6:**
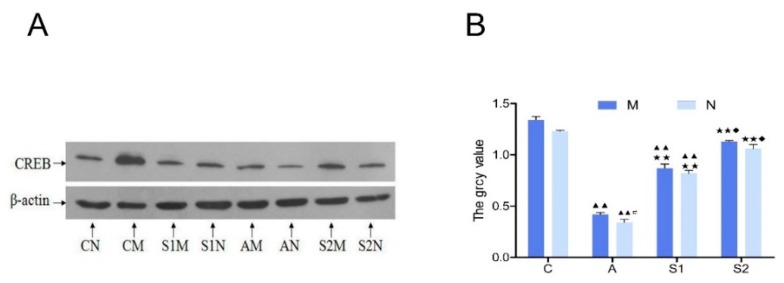
(**A**): Western blotting analysis for determining CREB protein expression levels in rat hippocampus. (**B**): Densitometric quantification of CREB protein expression (Mean ± SD; *n* = 10/group). The in-graph symbols indicate: ^▲▲^ *p* < 0.01 versus *Group C*; * *p* < 0.05 or ** *p* < 0.01 versus *Group A*; ^◆^ *p* < 0.05 versus *Group S1*; ^#^ *p* < 0.05 versus *Group M*.

**Figure 7:**
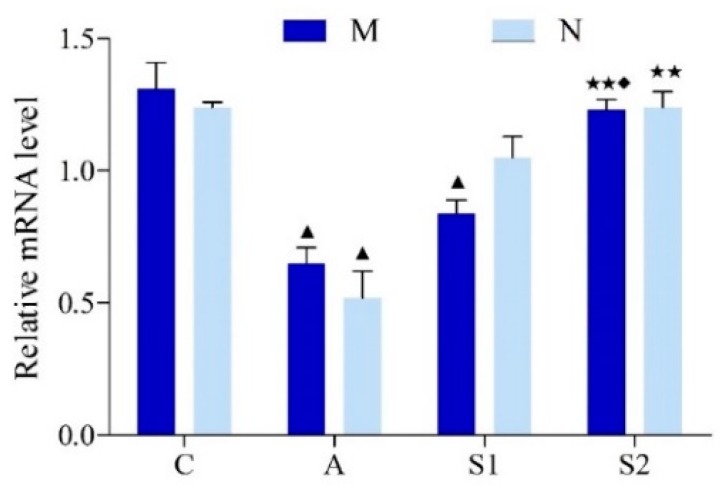
Changes of CREB mRNA expression in rat hippocampus. After the real-time PCR reactions were completed, the relative gene expressions were calculated using the 2^−ΔΔCT^ method. Data are presented as Mean ± SD (*n* = 10/group). The in-graph symbols indicate: ^▲^ *p* < 0.05 versus *Group C*; ** *p* < 0.01 versus *Group A*; ^◆^ *p* < 0.05 versus *Group S1*.

Hippocampal CREB protein expression in group A was significantly lower than the Control group (*p* < 0.01), indicating a strong inhibitory effect by DGA. On the other hand, both groups with swimming exercise training (S1 and S2) significantly increased the CREB expression (*p* < 0.01; Figure 6A,B). However, CREB protein level was not altered in the M subgroup under all treatment conditions, as compared with the N subgroups. Similarly, the level of hippocampal CREB mRNA expression in group A was significantly lower than all other groups, whereas CREB mRNA was significantly higher in groups C and S2 than other groups (*p* < 0.01; Figure 7).

### 3.7. CREB Gene Methylation Level in Hippocampus

**Figure 8:**
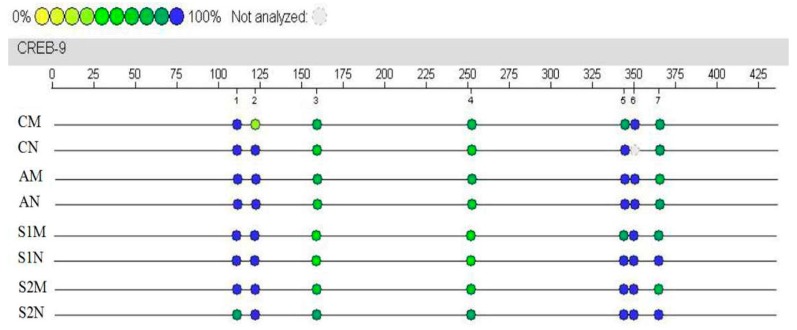
DNA methylation rate at the CpG site of *CREB* gene in rat hippocampus under various treatment conditions. The fragment containing nucleotide 56–470, with concentrated CpG sites, was selected as the target sequence. Abbreviated group names: CM—Saline-treated controls with Morris water maze training; CN—Saline-treated controls without Morris water maze training; AM—d-galactose injected rats received Morris water maze training; AN—d-galactose injected rats without Morris water maze training; S1M and S1N—Swimming exercise training before the d-galactose injection period with or without Morris water maze training; and S2M and S2N—Swimming exercise training during the d-galactose injection period with or without Morris water maze training.

**Figure 9:**
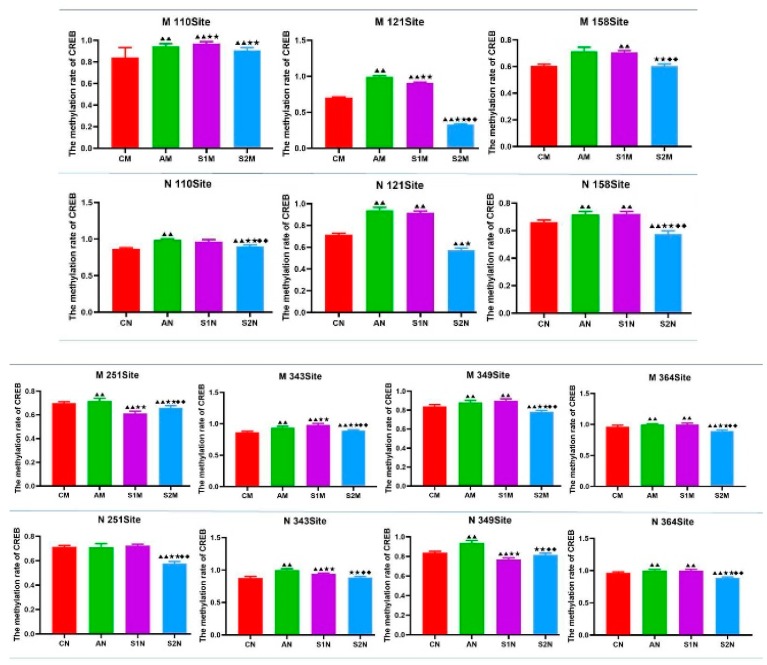
Comparison of DNA total methylation rate at each of the 7 sites of *CREB* gene in rat hippocampus under various treatment conditions. The fragment containing nucleotide 56–470, with concentrated CpG sites, was selected as the target sequence. Data are presented as Mean ± SD (*n* = 10/group). The in-graph symbols indicate: ^▲^ *p* < 0.05, ^▲▲^ *p* < 0.01 versus group CM or CN; * *p* < 0.05, ** *p* < 0.01 versus group AM or AN; and ^◆^ *p* < 0.05, ^◆◆^ *p* < 0.01 versus group S1M.

Group A had a relatively higher level of methylation, followed by Group S1. To the contrary, Group C and S2 had relatively low levels of methylation (Figure 8). Specifically, the highest overall methylation rate among the seven sites was the site 364, reaching 97.3%, followed by the sites 110, 343 and 349 as 93.2%, 93.1% and 86.6%, respectively (Figure 8). All of the 7 sites were significantly higher than the other sites (*p* < 0.01). Furthermore, Figure 9 shows the results in comparison of DNA methylation rate at each of the 7 sites of *CREB* gene in the rat hippocampus under various treatment conditions. The most notable findings are the reversal of the DGA-enhanced DNA methylation rate of CREB in the S2 group at several sites of CREB, i.e., site 121, 158, 251, 343, 349, and 364 (*p* < 0.01, S2M versus AM) in the subgroup M and site 110, 121, 158, 251, 343, 349, and 364 (*p* < 0.01, S2N versus AN) in the subgroup N (Figure 9). On the other hand, such beneficial effects were not consistently found in the S1 group (Figure 9).

## 4. Discussion

It has been well recognized that aging causes morphological changes in the brain, manifesting as decreased number of neurons, disorganized and atrophied neuronal cells, loose connective tissue, and reduced number of cellular organelles. The aging-induced degenerative morphological changes in hippocampal neurons may affect learning and memory function [20]. Our present study demonstrated that injections of d-galactose for six weeks (Group A) resulted in the above-mentioned typical aging-like functional changes and a salient finding of the present study is that swimming exercise training improved spatial learning and memory ability in drug-induced aging rats, assessed with the Morris water maze test, a classic neuroscience method in rodent studies [21]. The average escape latency was used as an important indicator for detecting spatial learning and memory in rats [22]. Our current study demonstrated that the average escape latency of rats gradually decreased when training times increased, and ultimately a stable spatial memory was formed. We found that while aging reduced spatial learning and memory ability, swimming exercise intervention either before or during d-galactose-induced aging had improved learning and memory in the rats, indicated by the significantly shorten escape latency in the exercise-trained groups on the third day of the Morris water maze test (Figure 2). However, such an exercise training-induced effect disappeared in the later stage of experiments (i.e., the fourth to sixth day). In addition, we observed that the space exploration ability (e.g., times of crossing platform and percentage of swim distance to original platform quadrant versus total distance) was significantly higher in group S2M as compared with groups AM and S1M (Figure 3). These results further suggest that the beneficial effect of exercise intervention varies with the timing of exercise intervention. Exercise training during the aging process had the most significant benefits, whereas exercise before the drug-induced aging had essentially no impact. The cellular mechanism underlying this clear dependence on the protective time window of exercise training requires further in-depth investigation.

The synapse is extremely sensitive to various stimuli and synaptic plasticity refers to the ability of the synapse to adapt its structure and function to a variety of stimuli and synaptic plasticity has been considered as the neurobiological basis for learning and memory formation, whereas both synaptophysin (Syp) and metabotropic glutamate receptor 1 (mGluR1) are used as the most representative indexes for detection of synaptic plasticity [23,24], which are usually down-regulated during aging [25,26]. Since Syp is a synaptic vesicle-specific membrane channel, a reduction in its numbers would lead to a reduced transport capacity of the synaptic vesicle that blocks synaptic transmission and in turn negatively affects the transfer, processing and storage of the nervous system information. Similarly, because mGluR1 cells are associated with a variety of second messengers, a reduction in GluR1 content may impede the downstream signaling pathways mediated by GluR1.

Closely relevant to the current study, a few previous studies reported that exercise training altered expression levels of Syp and mGluR1 in aging tissues. For examples, Chen et al. found that moderate-intensity physical training enhanced hippocampal Syp levels that were decreased by aging [27]. A recent report showed that voluntary exercise significantly increased Syp expression after focal cerebral ischemia in rats [28]. Similarly, it was demonstrated that after focal cerebral ischemia, early motor balance and coordination training significantly increased synaptophysin expression in the ischemic hippocampus and improved neurological function recovery [29]. The results from our current study suggest that Syp showed a lamellar distribution and three bands were clearly visible in the dentate gyrus with the expression in the cytoplasm. The distribution of mGluR1 was spotty, but apparent on the cell membrane. The sub-acute aging process induced by d-galactose injection resulted in significant decrease in the contents of Syp and mGluR1 as compared with the control group (Figure 5). The expression levels of Syp and mGluR1 increased in all exercisetrained groups with higher significance in Group S2, indicating that aerobic exercise with water maze training during the aging process was more effective in improving Syp, and in turn learning and memory. Interestingly, exercise training before the drug-induced aging in Group S1 did not restore mGluR1 expression (Figure 5).

Furthermore, activation of mGluR1/5 promotes the generation of cAMP via activation of protein kinase A (PKA), which phosphorylates CREB at its N-terminal domain leading to activation of CREB that regulates the downstream gene transcription and protein synthesis associated with learning and memory. Previous studies have shown that the cAMP/PKA/CREB signaling pathway directly involved in learning and memory formation [30,31]. CREB is an in-eukaryotic nuclear protein that regulates gene transcription and is widely distributed in the cerebral cortex and hippocampus and plays a key regulator role in signal transduction in the central nervous system and controls nerve cell cycles, induction and differentiation of neurons, and modulating synaptic plasticity [30,32]. CREB also plays a key role in synaptic long-term potentiation induction and maintenance [33]. Furthermore, it was demonstrated that CREB could activate BDNF transcription, improve long-term memory ability in rats, and was closely related with spatial learning and memory [34,35]. Takeo et al. previously showed that CREB was directly involved in water maze spatial learning and memory formation [31]. Our present study revealed that hippocampal CREB protein and mRNA expression was decreased in aging rat hippocampus as compared with the Control group ( Figure 6; Figure 7). Similarly, the aging-caused decrease in CREB expression was significantly restored in both group S1 and S2, whereas the restoration level of CREB was more significant in group S2 than those in group S1, suggesting that exercise training during the aging process was more effective for maintaining CREB protein and mRNA expression in the aging rat hippocampus (Figure 6 and Figure 7). These results suggest that the absence of exercise during the organ senescence process would reduce its neuroprotective efficacy and therefore exercise should be carried out as soon as possible during the aging process on a continuous basis.

Finally, our present study revealed that exercise training reduced *CREB* gene methylation level in the drug-induced aging rat hippocampus (Figure 8). DNA methylation is a major epigenetic modification that can modulate gene function without altering the gene sequence [36]. DNA methylation may turn off certain gene activities, while de-methylation may induce or re-activate the gene [37]. A previous study by Horvath et al. suggested that aging may hyper-methylate certain genes in vivo, thus leading to transcriptional silencing, thereby causing a series of degenerative changes in cells that lead to aging-related diseases in various organ systems [38], including the age-dependent epigenetic modulation of gene expression in human skeletal muscles [39,40]. Interestingly, Gomez-Pinilla et al. reported that exercise training a running wheel can reduce DNA methylation level in the BDNF promoter domain, thus enhancing BDNF transcription to improve learning and cognition in adult rats [41]. Similarly, Aguiar et al. demonstrated in aged rats that short bouts of mild-intensity exercise improve spatial learning and memory and they demonstrated the involvement of hippocampal plasticity via Akt, CREB and BDNF signaling [42]. Therefore, the new results of our present study further support the notion that drug-induced aging causes hypermethylation of CREB in hippocampus and in turn results in learning and memory dysfunction. As shown in Figure 8, hippocampal CREB methylation rates in the exercise-trained groups were significantly lower than the drug-induced aging rats and such a de-methylation and reactivation of the key genes may effectively reduce loss of function due to transcriptional silencing and dysfunction of certain downstream gene targets. These findings suggest that exercise training during the aging may effectively modify CREB expression that allows to complete RNA transcription and protein translation, and subsequently improve hippocampal learning and memory capacity. However, we found that exercise training before aging did not show similar protective effects, or to the opposite, their CREB methylation rate was even higher than the aging alone group. There is a possibility that in the aged animals, relatively high-intensity of exercise may not lead to improvement of degenerative changes in the neuronal cells, but rather caused greater damage and accelerated aging in the neurons.

Nevertheless, the present study has several limitations. First, the findings of our current study in the DGA-induced aging-like rats should be further validated in in future studies using naturally aged rats to enhance the physiological relevance. Second, we employed only a single behavioral test to evaluate learning and memory of the rats. Future studies should use multiple tests to verify the consistency of the findings on learning and memory function. Third, there is a general consensus that neuroprotection induced by physical exercise is a multi-factorial phenomenon involving divergent cellular and molecular mechanisms, such as BDNF, angiogenesis and neurogenesis, which had not been assessed simultaneously in the present study due to the main focus and scope of the present investigation. Forth, we used only male rats in the current study and we cannot determine if there are possible differences in the DGA-induced aging as well as the physiological and cellular response to swimming exercise training among male and female rats. Finally, fifth, we only measured mRNA, total protein and methylation levels of CREB, without examining the phosphorylation levels of CREB in the hippocampal samples. It is notable that recent study demonstrated that spatial memory exercise changes the expression levels of phosphorylated CREB, which is an important signaling mediator for learning and memory process [43]. Therefore, future in-depth studies are warranted to evaluate the potential contributions of the multiple neuroprotective mechanisms in swimming exercise-induced functional improvement in learning and memory in aged individuals.

## 5. Conclusions

The present study demonstrated that 6-week daily swimming exercise training during the aging process, as compared with exercise training before the aging progression, was more effective than in protecting hippocampal neurons against DGA-induced degenerative changes and in turn maintained synaptic plasticity, possibly via reactivation/upregulation of CREB mRNA and protein, inhibition of DGA-enhanced CREB methylation, and consequently alleviated DGA-induced functional impairment in learning and memory.

## References

[1] Donzis E.J., Tronson N.C. (2014). Modulation of learning and memory by cytokines: Signaling mechanisms and long term consequences. Neurobiol. Learn. Mem..

[2] Molfese D.L. (2011). Advancing neuroscience through epigenetics: Molecular mechanisms of learning and memory. Dev. Neuropsychol..

[3] Kennard J.A., Woodruff-Pak D.S. (2011). Age sensitivity of behavioral tests and brain substrates of normal aging in mice. Front. Aging Neurosci..

[4] Inoue K., Hanaoka Y., Nishijima T., Okamoto M., Chang H., Saito T., Soya H. (2015). Long-term mild exercise training enhances hippocampus-dependent memory in rats. Int. J. Sports Med..

[5] Suwabe K., Byun K., Hyodo K., Reagh Z.M., Roberts J.M., Matsushita A., Saotome K., Ochi G., Fukuie T., Suzuki K. (2018). Rapid stimulation of human dentate gyrus function with acute mild exercise. Proc. Natl. Acad. Sci. USA.

[6] Nishijima T., Torres-Aleman I., Soya H. (2016). Exercise and cerebrovascular plasticity. Prog. Brain Res..

[7] Sadigh-Eteghad S., Majdi A., McCann S.K., Mahmoudi J., Vafaee M.S., Macleod M.R. (2017). d-galactose-induced brain ageing model: A systematic review and meta-analysis on cognitive outcomes and oxidative stress indices. PLoS ONE.

[8] Wei H., Li L., Song Q., Ai H., Chu J., Li W. (2005). Behavioural study of the d-galactose induced aging model in C57BL/6J mice. Behav. Brain Res..

[9] Chae C.H., Kim H.T. (2009). Forced, moderate-intensity treadmill exercise suppresses apoptosis by increasing the level of NGF and stimulating phosphatidylinositol 3-kinase signaling in the hippocampus of induced aging rats. Neurochem. Int..

[10] Li L., Xu M., Shen B., Li M., Gao Q., Wei S.G. (2016). Moderate exercise prevents neurodegeneration in d-galactose-induced aging mice. Neural Regen. Res..

[11] Yu F., Xu B., Song C., Ji L., Zhang X. (2013). Treadmill exercise slows cognitive deficits in aging rats by antioxidation and inhibition of amyloid production. Neuroreport.

[12] Berchicci M., Lucci G., Di R.F. (2013). Benefits of physical exercise on the aging brain: The role of the prefrontal cortex. J. Gerontol. A Biol. Sci. Med. Sci..

[13] Gomez-Pinilla F., Hillman C. (2013). The influence of exercise on cognitive abilities. Compr. Physiol..

[14] Lautenschlager N.T., Cox K., Cyarto E.V. (2012). The influence of exercise on brain aging and dementia. Biochim. Biophys. Acta.

[15] Baptista S., Piloto N., Reis F., Teixeira-de-Lemos E., Garrido A.P., Dias A., Lourenço M., Palmeiro A., Ferrer-Antunes C., Teixeira F. (2008). Treadmill running and swimming imposes distinct cardiovascular physiological adaptations in the rat: Focus on serotonergic and sympathetic nervous systems modulation. Acta Physiol. Hung..

[16] Geenen D., Buttrick P., Scheuer J. (1988). Genetic and epigenetic factors are associated with expression of respiratory chain component NDUFB6 in human skeletal muscle. J. Appl. Physiol..

[17] Contarteze R.V., Manchado Fde B., Gobatto C.A., De Mello M.A. (2008). Stress biomarkers in rats submitted to swimming and treadmill running exercises. Comp. Biochem. Physiol. A Mol. Integr. Physiol..

[18] Chen C.F., Lang S.Y., Zuo P.P., Yang N., Wang X.Q., Xia C. (2006). Effects of d-galactose on the expression of hippocampal peripheral-type benzodiazepine receptor and spatial memory performances in rats. Psychoneuroendocrinology.

[19] Pierozan P., Jerneren F., Ransome Y., Karlsson O. (2017). The choice of euthanasia method affects metabolic serum biomarkers. Basic Clin. Pharmacol. Toxicol..

[20] Lombroso P.J., Ogren M.P. (2008). Learning and memory, part I: Brain regions involved in two types of learning and memory. J. Am. Acad. Child. Adolesc. Psychiatry.

[21] Morris R. (1984). Developments of a water-maze procedure for studying spatial learning in the rat. J. Neurosci. Methods.

[22] Anderson E.M., Moenk M.D., Barbaro L., Clarke D.A., Matuszewich L. (2013). Effects of pretraining and water temperature on female rats’ performance in the Morris water maze. Physiol. Behav..

[23] Mameli M., Halbout B., Creton C., Engblom D., Parkitna J.R., Spanagel R., Luscher C. (2009). Cocaine-evoked synaptic plasticity: Persistence in the VTA triggers adaptations in the NAc. Nat. Neurosci..

[24] Yang J., Yao Y., Wang L., Yang C., Wang F., Guo J., Wang Z., Yang Z., Ming D. (2017). Gastrin-releasing peptide facilitates glutamatergic transmission in the hippocampus and effectively prevents vascular dementia induced cognitive and synaptic plasticity deficits. Exp. Neurol..

[25] Bartolome M.V., Zuluaga P., Carricondo F., Gil-Loyzaga P. (2009). Immunocytochemical detection of synaptophysin in C57BL/6 mice cochlea during aging process. Brain Res. Rev..

[26] Simonyi A., Ngomba R.T., Storto M., Catania M.V., Miller L.A., Youngs B., Di Giorgi-Gerevini V., Nicoletti F., Sun G.Y. (2005). Expression of groups I and II metabotropic glutamate receptors in the rat brain during aging. Brain Res..

[27] Chen Y.C., Chen Q.S., Lei J.L., Wang S.L. (1998). Physical training modifies the age-related decrease of GAP-43 and synaptophysin in the hippocampal formation in C57BL/6J mouse. Brain Res..

[28] Nie J., Yang X., Tang Q., Shen Q., Li S. (2016). Willed-movement training reduces brain damage and enhances synaptic plasticity related proteins synthesis after focal ischemia. Brain Res. Bull..

[29] Seo H.G., Kim D.Y., Park H.W., Lee S.U., Park S.H. (2010). Early motor balance and coordination training increased synaptophysin in subcortical regions of the ischemic rat brain. J. Korean Med. Sci..

[30] Cammarota M., Bevilaqua L.R., Ardenghi P., Paratcha G., de Stein M.L., Izquierdo I., Medina J.H. (2000). Learning-associated activation of nuclear MAPK, CREB and Elk-1, along with Fos production, in the rat hippocampus after a one-trial avoidance learning: Abolition by NMDA receptor blockade. Mol. Brain Res..

[31] Takeo S., Niimura M., Miyake-Takagi K., Nagakura A., Fukatsu T., Ando T., Takagi N., Tanonaka K., Hara J. (2003). A possible mechanism for improvement by a cognition-enhancer nefiracetam of spatial memory function and cAMP-mediated signal transduction system in sustained cerebral ischaemia in rats. Br. J. Pharmacol..

[32] Waltereit R., Weller M. (2003). Signaling from cAMP/PKA to MAPK and synaptic plasticity. Mol. Neurobiol..

[33] Alzoubi K.H., Alkadhi K.A. (2014). Levothyroxin replacement therapy restores hypothyroidism induced impairment of L-LTP induction: Critical role of CREB. Brain Res. Bull..

[34] Dubnau J., Tully T. (1998). Gene discovery in Drosophila: New insights for learning and memory. Annu. Rev. Neurosci..

[35] Martin K.C., Michael D., Rose J.C., Barad M., Casadio A., Zhu H., Kandel E.R. (1997). MAP kinase translocates into the nucleus of the presynaptic cell and is required for long-term facilitation in Aplysia. Neuron.

[36] Dahl C., Guldberg P. (2003). DNA methylation analysis techniques. Biogerontology.

[37] Ehrlich M. (2002). DNA methylation in cancer: Too much, but also too little. Oncogene.

[38] Horvath S., Zhang Y., Langfelder P., Kahn R.S., Boks M.P., van E.K., van den Berg L.H., Ophoff R.A. (2012). Aging effects on DNA methylation modules in human brain and blood tissue. Genome Biol..

[39] Ling C., Poulsen P., Simonsson S., Ronn T., Holmkvist J., Almgren P., Hagert P., Nilsson E., Mabey A.G., Nilsson P. (2007). Genetic and epigenetic factors are associated with expression of respiratory chain component NDUFB6 in human skeletal muscle. J. Clin. Investig..

[40] Ronn T., Poulsen P., Hansson O., Holmkvist J., Almgren P., Nilsson P., Tuomi T., Isomaa B., Groop L., Vaag A. (2008). Age influences DNA methylation and gene expression of COX7A1 in human skeletal muscle. Diabetologia.

[41] Gomez-Pinilla F., Zhuang Y., Feng J., Ying Z., Fan G. (2011). Exercise impacts brain-derived neurotrophic factor plasticity by engaging mechanisms of epigenetic regulation. Eur. J. Neurosci..

[42] Aguiar A.S., Castro A.A., Moreira E.L., Glaser V., Santos A.R., Tasca C.I., Latini A., Prediger R.D. (2011). Short bouts of mild-intensity physical exercise improve spatial learning and memory in aging rats: Involvement of hippocampal plasticity via AKT, CREB and BDNF signaling. Mech. Ageing Dev..

[43] Subbanna S., Joshi V., Basavarajappa B.S. (2018). Activity-dependent signaling and epigenetic abnormalities in mice exposed to postnatal ethanol. Neuroscience.

